# Testicular biopsies microarray analysis reveals circRNAs are involved in the pathogenesis of non-obstructive azoospermia

**DOI:** 10.18632/aging.102765

**Published:** 2020-02-06

**Authors:** Hao Bo, Zhizhong Liu, Ruiling Tang, Guanghui Gong, Xingming Wang, Han Zhang, Fang Zhu, Dai Zhou, Wenbing Zhu, Yueqiu Tan, Liqing Fan

**Affiliations:** 1 Institute of Reproductive and Stem Cell Engineering, School of Basic Medical Science, Central South University, Changsha, China; 2 Reproductive and Genetic Hospital of CITIC-Xiangya, Changsha, China; 3 Hunan Cancer Hospital and The Affliated Cancer Hospital of Xiangya School of Medicine, Central South University, Changsha, China

**Keywords:** circRNAs, expression profile, non-obstructive azoospermia (NOA), miRNAs, ceRNA network

## Abstract

Circular RNAs (circRNAs) have been reported to be involved in many diseases. But there is no report on circRNAs in non-obstructive azoospermia (NOA). The purpose of this paper is to explore the circular RNA expression profile and potential functions of circRNAs in NOA patients. We first preformed circRNA expression profiling analysis using a circRNA microarray in testicular samples from NOA and obstructive azoospermia (OA) patients. CircRNAs were validated by qRT-PCR. Bioinformatics analysis were used to construct the ceRNA network. GO and KEGG enrichment analysis were performed by using DAVID. Microarray analysis identified 82 differentially expressed circRNAs in NOA specimens. The differential expression of hsa_circRNA_402130, hsa_circRNA_072697, hsa_circRNA_030050, hsa_circRNA_100812 and hsa_circRNA_406168 was confirmed by qRT-PCR. Enrichment analysis revealed the association of hsa_circRNA_402130 and hsa_circRNA_072697 with multiple signaling pathways. The data indicated that circRNAs were significantly dysregulated in NOA specimens and might involve in the pathogenesis of NOA.

## INTRODUCTION

Circular RNAs (circRNAs) are a class of closed circular RNA molecules that rarely encode proteins and have no 5 ‘caps and 3’ poly(A) tails. Their unique characteristics, such as better stability and resistance to nucleases make circRNAs potential candidates for clinical diagnostic/prognostic biomarkers [1]. The molecular mechanisms of circRNAs are diverse. In addition to acting as miRNA sponges, they may also play a role by binding protein or DNA sequences [2–4]. Some of them can also regulate gene expression by affecting RNA splicing or translating into short peptides [5]. They are conserved among different species, and also exhibit tissue-specific and developmental-stage specific traits [6]. Emerging evidences suggest that circRNAs are likely to play important function in different physiological or pathological processes. A multitude of studies have demonstrated that circRNAs are associated with various diseases, including cancer, Alzheimer’s disease, ovarian endometriosis, preeclampsia [7–11]. Moreover, a previous study has reported that there are 15,996 circRNAs in normal human testis and more than 20 testis-derived circRNAs are stably expressed in seminal plasma [5]. All the above evidences indicate that circRNAs might play a role in spermatogenesis and maturation. However, the global expression and function of circRNAs in non-obstructive azoospermia (NOA) are largely unknown.

NOA is considered a major cause of male infertility [12, 13], which can’t be completely cured by assisted reproductive technology (ART). Several genetic and epigenetic factors, especially Y chromosome microdeletion, are associated with the pathogenesis NOA [14–18]. In recent years, genome-wide association analysis (GWAS), has led to the identification of a few key NOA risk loci including PRMT6, PEX10, HLA-DRA [14, 16]. In addition to these genetic factors, epigenetic factors also play an important role in the development of NOA. For instance, Chencheng Yao et al. found that there were many differentially expressed miRNAs in testicular spermatogonia, pachytene spermatocytes, and round spermatids between NOA and OA patients [13]. Further, another group also found that miRNA-188-3p could regulate spermatogenic cell apoptosis by targeting MLH1 [19]. However, the epigenetic regulatory mechanisms, especially the role of circRNAs, in NOA are yet unknown.

Hence, the purpose of this study was to elucidate the expression profile of circRNAs and unravel the possible functions and interactions of circRNAs in NOA.

## RESULTS

### Identification of the dysregulated circRNAs between NOA and OA groups

**Figure 1:**
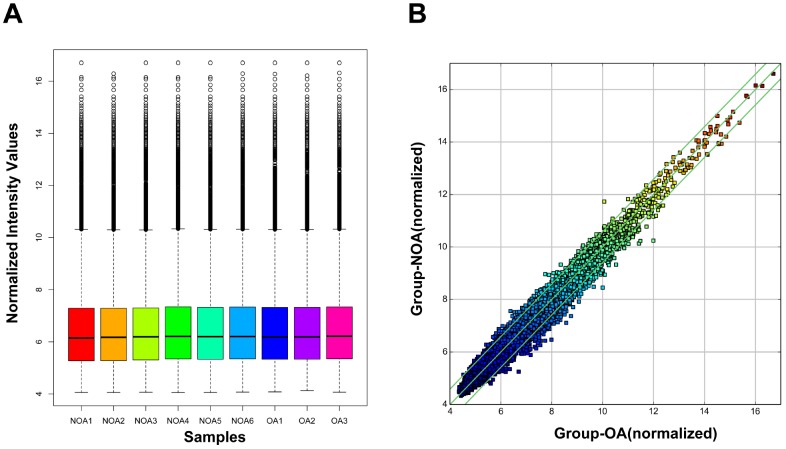
**Box plot and scatter plot of circRNA signal value variation in OA and NOA samples.** (**A**) The distribution of circRNAs for the nine samples. (OA: obstructive azoospermia; NOA: non-obstructive azoospermia). (**B**) Scatter plot showed variations in circRNA expression between OA and NOA samples. CircRNAs located above the top green line and below the bottom green line were more than 1.5-fold between OA and NOA samples.

**Figure 2:**
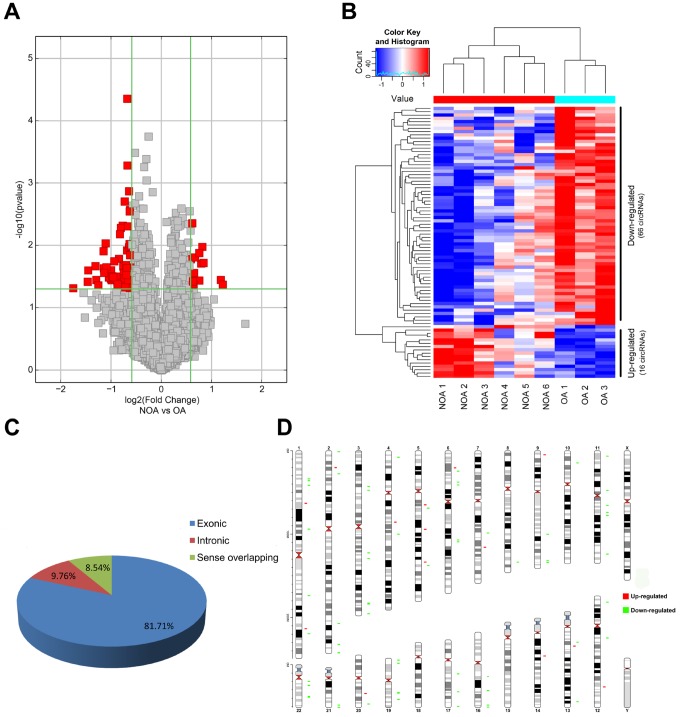
**Characterization of differentially expressed circRNA in OA and NOA samples.** (**A**) The volcano plots display the differential circRNAs expression between the two groups. Red points denote the differentially expressed circRNAs with statistical significance. (fold change ≥1.5 and p <0.05). (**B**) Heatmap shows the 16 up-regulated circRNAs and 66 down-regulated circRNAs in NOA. (**C**) The pie charts show the origin of transcription of differentially expressed circRNAs. (**D**) The chromosomal location of differentially expressed circRNAs is shown in the last figure. Red: up-regulated circRNAs in NOA; green: down-regulated circRNAs in NOA.

We performed microarray-based profiling analyses to screen the differentially expressed circRNAs in 6 NOA and 3 OA tissues. The box plot depicts similar distributions of the intensities from all the normalized datasets (Figure 1A). Raw variation of circRNA expression between the two groups is shown in the scatter plot (Figure 1B). Volcano plots and hierarchical clustering exhibit the differentially expressed circRNAs between the NOA and OA samples (Figure 2A and 2B). Using a threshold of fold change (FC) ≥ 1.5 and a p-value < 0.05, we identified 82 differentially expressed circRNAs, of which 16 were up-regulated and 66 were down-regulated in NOA tissues compared with OA tissues (Table 1 and Table 2). According to the annotation of human circRNAs, we found 81.71% of the dysregulated circRNAs were exonic circRNAs, 9.76% were intronic circRNAs and 8.54% were sense overlapping circRNAs (Figure 2C). Further analysis revealed that these dysregulated circRNAs were mostly distributed on chr1, chr2, chr3, chr4, chr5, chr6, chr7, chr8, chr9, chr10, chr11, chr12, chr13, chr14, chr16, chr17, chr19, chr20, chr21 and chr22, but not on chr15, chr18, chrX and chrY (Figure 2D).

**Table 1:** Significantly upregulated circRNAs in NOA.

**Significantly upregulated circRNAs**					
**probeID**	**P-value**	**FDR**	**FC (abs)**	**Regulation**	**circRNA**
ASCRP3001555	0.0461916	0.957391589	1.52275	up	hsa_circRNA_401590
ASCRP3002427	0.016655094	0.957391589	1.5038532	up	hsa_circRNA_002032
ASCRP3002765	0.035853368	0.957391589	2.2698958	up	hsa_circRNA_072697
ASCRP3003870	0.010571567	0.957391589	1.7599646	up	hsa_circRNA_101130
ASCRP3004000	0.03573483	0.957391589	1.5243845	up	hsa_circRNA_100452
ASCRP3006656	0.019589109	0.957391589	1.7348182	up	hsa_circRNA_103916
ASCRP3007758	0.028997996	0.957391589	1.5351706	up	hsa_circRNA_070294
ASCRP3007963	0.019122487	0.957391589	1.7931198	up	hsa_circRNA_001512
ASCRP3009230	0.027775787	0.957391589	1.5701942	up	hsa_circRNA_031757
ASCRP3009252	0.004415576	0.957391589	1.5314721	up	hsa_circRNA_104078
ASCRP3009351	0.01558938	0.957391589	1.5958358	up	hsa_circRNA_073942
ASCRP3010355	0.013282587	0.957391589	1.6874562	up	hsa_circRNA_030050
ASCRP3010888	0.036692735	0.957391589	1.6901888	up	hsa_circRNA_103075
ASCRP3011370	0.042166787	0.957391589	1.5615877	up	hsa_circRNA_406996
ASCRP3011901	0.042668697	0.957391589	2.3337021	up	hsa_circRNA_001506
ASCRP3012974	0.037931142	0.957391589	1.5484401	up	hsa_circRNA_002006

**Table 2:** Significantly downregulated circRNAs in NOA

**Significantly downregulated circRNAs**					
**probeID**	**P-value**	**FDR**	**FC (abs)**	**Regulation**	**circRNA**
ASCRP3000334	0.016382909	0.957391589	1.7917313	down	hsa_circRNA_403874
ASCRP3000537	0.004831071	0.957391589	1.6995075	down	hsa_circRNA_001937
ASCRP3000663	0.021040239	0.957391589	1.6627497	down	hsa_circRNA_406402
ASCRP3000825	0.002705609	0.957391589	1.5361052	down	hsa_circRNA_406267
ASCRP3000954	0.005248263	0.957391589	1.746061	down	hsa_circRNA_402130
ASCRP3000991	0.037238178	0.957391589	1.730753	down	hsa_circRNA_406822
ASCRP3001155	0.045260941	0.957391589	1.536961	down	hsa_circRNA_029998
ASCRP3001768	0.025224767	0.957391589	2.7399933	down	hsa_circRNA_406168
ASCRP3001923	0.048642805	0.957391589	3.3645337	down	hsa_circRNA_025349
ASCRP3002888	0.049366594	0.957391589	1.6916213	down	hsa_circRNA_402150
ASCRP3002953	0.012038305	0.957391589	1.6022409	down	hsa_circRNA_406503
ASCRP3003279	0.036135037	0.957391589	1.9818485	down	hsa_circRNA_406775
ASCRP3003518	0.035892518	0.957391589	2.4505456	down	hsa_circRNA_101053
ASCRP3003843	0.018020351	0.957391589	1.9115564	down	hsa_circRNA_406194
ASCRP3003942	0.00052197	0.957391589	1.5953273	down	hsa_circRNA_406419
ASCRP3003975	0.009862311	0.957391589	1.5153189	down	hsa_circRNA_103161
ASCRP3004118	0.036664644	0.957391589	1.5911543	down	hsa_circRNA_103141
ASCRP3004393	0.026087538	0.957391589	1.5282233	down	hsa_circRNA_067007
ASCRP3004478	0.010656101	0.957391589	1.5616605	down	hsa_circRNA_102558
ASCRP3004662	0.033021538	0.957391589	1.7230277	down	hsa_circRNA_000780
ASCRP3004702	0.022526843	0.957391589	1.5259744	down	hsa_circRNA_015279
ASCRP3004757	0.012437973	0.957391589	2.2150382	down	hsa_circRNA_104935
ASCRP3005255	0.047336724	0.957391589	1.5978168	down	hsa_circRNA_007328
ASCRP3005451	0.001349981	0.957391589	1.5599409	down	hsa_circRNA_103867
ASCRP3005694	0.04141577	0.957391589	1.6261871	down	hsa_circRNA_101894
ASCRP3005793	0.031346564	0.957391589	1.6079474	down	hsa_circRNA_102966
ASCRP3006067	0.009529304	0.957391589	1.5492371	down	hsa_circRNA_103348
ASCRP3006096	0.038650961	0.957391589	1.5329346	down	hsa_circRNA_023576
ASCRP3006334	0.010459305	0.957391589	1.5086231	down	hsa_circRNA_400185
ASCRP3006335	0.023372267	0.957391589	2.2086086	down	hsa_circRNA_404655
ASCRP3006336	0.046143308	0.957391589	1.8350186	down	hsa_circRNA_104940
ASCRP3006936	0.025160274	0.957391589	1.9992925	down	hsa_circRNA_102166
ASCRP3007419	0.025860647	0.957391589	1.5221658	down	hsa_circRNA_006604
ASCRP3007490	4.4193E-05	0.587369562	1.5977581	down	hsa_circRNA_406768
ASCRP3007702	0.047580725	0.957391589	1.5580697	down	hsa_circRNA_000883
ASCRP3007956	0.034250275	0.957391589	2.0042991	down	hsa_circRNA_006710
ASCRP3008513	0.032785099	0.957391589	2.0605802	down	hsa_circRNA_403457
ASCRP3008758	0.022383408	0.957391589	2.1321055	down	hsa_circRNA_400696
ASCRP3008931	0.035547528	0.957391589	1.7530169	down	hsa_circRNA_001695
ASCRP3009069	0.014371347	0.957391589	1.5358115	down	hsa_circRNA_103601
ASCRP3009323	0.046773984	0.957391589	1.6990883	down	hsa_circRNA_100166
ASCRP3009406	0.027542185	0.957391589	2.1979834	down	hsa_circRNA_102232
ASCRP3009440	0.031767553	0.957391589	1.7600919	down	hsa_circRNA_007624
ASCRP3009894	0.021930582	0.957391589	1.6907468	down	hsa_circRNA_002141
ASCRP3009982	0.009261095	0.957391589	2.1517926	down	hsa_circRNA_002796
ASCRP3010075	0.006608806	0.957391589	1.7673139	down	hsa_circRNA_051123
ASCRP3010266	0.042141617	0.957391589	1.6013384	down	hsa_circRNA_103181
ASCRP3010752	0.049588744	0.957391589	1.531321	down	hsa_circRNA_405511
ASCRP3011101	0.031160151	0.957391589	1.9640111	down	hsa_circRNA_059085
ASCRP3011253	0.020745727	0.957391589	1.6280942	down	hsa_circRNA_003145
ASCRP3011607	0.027895917	0.957391589	1.6782159	down	hsa_circRNA_092478
ASCRP3011736	0.039612091	0.957391589	1.7179174	down	hsa_circRNA_407124
ASCRP3011751	0.023198686	0.957391589	1.6108817	down	hsa_circRNA_100812
ASCRP3011979	0.048109527	0.957391589	1.5443097	down	hsa_circRNA_008142
ASCRP3012222	0.047250017	0.957391589	1.6431699	down	hsa_circRNA_001752
ASCRP3012384	0.032114065	0.957391589	1.9400004	down	hsa_circRNA_403560
ASCRP3013001	0.021611589	0.957391589	2.4807294	down	hsa_circRNA_003671
ASCRP3013007	0.002843475	0.957391589	1.5463175	down	hsa_circRNA_102552
ASCRP3013008	0.001979569	0.957391589	1.6561938	down	hsa_circRNA_102551
ASCRP3013058	0.033675352	0.957391589	1.6553273	down	hsa_circRNA_406350
ASCRP3013126	0.043940509	0.957391589	2.3814219	down	hsa_circRNA_402731
ASCRP3013133	0.035888866	0.957391589	1.5808639	down	hsa_circRNA_000328
ASCRP3013332	0.004952971	0.957391589	1.5817435	down	hsa_circRNA_104168
ASCRP3013372	0.042368459	0.957391589	1.8241931	down	hsa_circRNA_068259
ASCRP3013501	0.029805804	0.957391589	1.6140515	down	hsa_circRNA_100900
ASCRP3013569	0.038372041	0.957391589	2.7556443	down	hsa_circRNA_100981

### Validation of circRNAs using qRT-PCR

**Figure 3:**
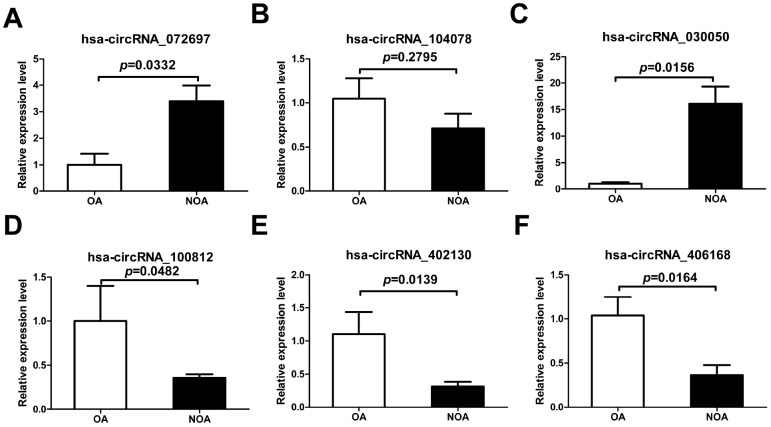
**Confirmation of the six candidate circRNAs by qRT-PCR.** All RNAs of 6 NOA and 3 OA patients were included**.** The experiments were repeated three times. 2-ΔΔCt method was used to measure gene expression. The values are presented as the mean ± SE.

To further confirm the results of circRNA microarray, we chose six dysregulated circRNAs at random, including three down-regulated (hsa_circRNA_402130, hsa_circRNA_100812 and hsa_circRNA_406168) and three up-regulated (hsa_circRNA_072697, hsa_circ RNA_104078 and hsa_circRNA_030050). qRT-PCR in NOA and OA samples showed that the expression of hsa_circRNA_072697, hsa_circRNA_402130, hsa_ circRNA_100812, hsa_circRNA_030050 and hsa_circ RNA_406168 was consistent with the microarray data. And the difference in expression of hsa_circ RNA_072697, hsa_circRNA_030050, hsa_circRNA_ 100812, hsa_circRNA_402130 and hsa_circRNA_406168 was statistically significant (Figure 3A, 3C, 3D, 3E, 3F, p=0.0332, p=0.0156, p=0.0482, p=0.0139, p=0.0164). The expression of hsa_circRNA_104078, however, did not follow the microarray and was not statistically significant (Figure 3B, p=0.2795).

### MicroRNA response elements analysis of validated circRNAs

**Figure 4:**
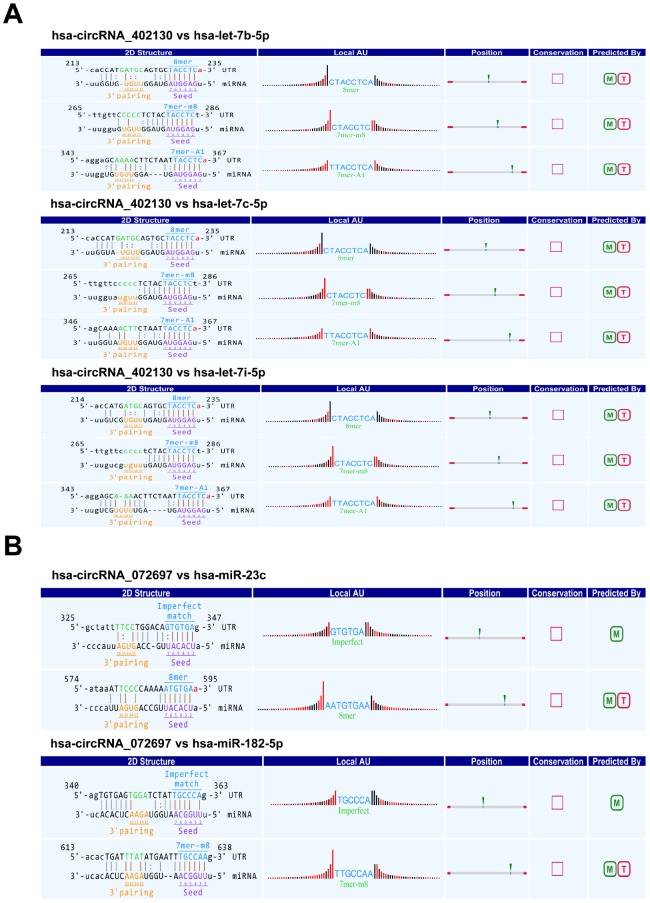
**Prediction of circRNA/miRNA interactions by Arraystar’s homemade miRNA target prediction software based on TargetScan and miRanda.** (**A**) The potential miRNA targets of hsa_circRNA_402130. (**B**) The potential miRNA targets of hsa_circRNA_072697. Local AU: Schematic diagram of the AU weights on both sides of the seed site. The empty square under “Conservation” indicates the lack of conserved information of circRNA between the species. M: miRanda; T: TargetScan.

Several studies have demonstrated that circRNAs regulate the expression of their target genes by competing with their target miRNAs for binding [8, 20, 21]. The Arraystar’s homemade miRNA target prediction software based on TargetScan and miRanda was utilized to predict the circRNA-microRNA interactions of two confirmed circRNAs (hsa_circRNA_402130 and hsa_circRNA_ 072697). The top 5 targeted miRNAs were then identified based on the miRNA support vector regression (mirSVR) scores. Studies have shown that some NOA-related miRNAs are dysregulated in the testicular tissues of patients with NOA [22, 23]. So we selected the the potential miRNAs shared by the software and the reports to construct circRNA–miRNA interactions. The 2D structure was created from the sequence analysis of microRNA response elements (MREs) (Figure 4A and 4B). For hsa_circRNA_402130, the potential miRNA targets included hsa-let-7b-5p, hsa-let-7c-5p and hsa-let-7i-5p, and for hsa_circRNA_072697 the miRNA included hsa-miR-23c and hsa-miR-182-5p.

### CircRNA-miRNA-mRNA interaction analysis

**Figure 5:**
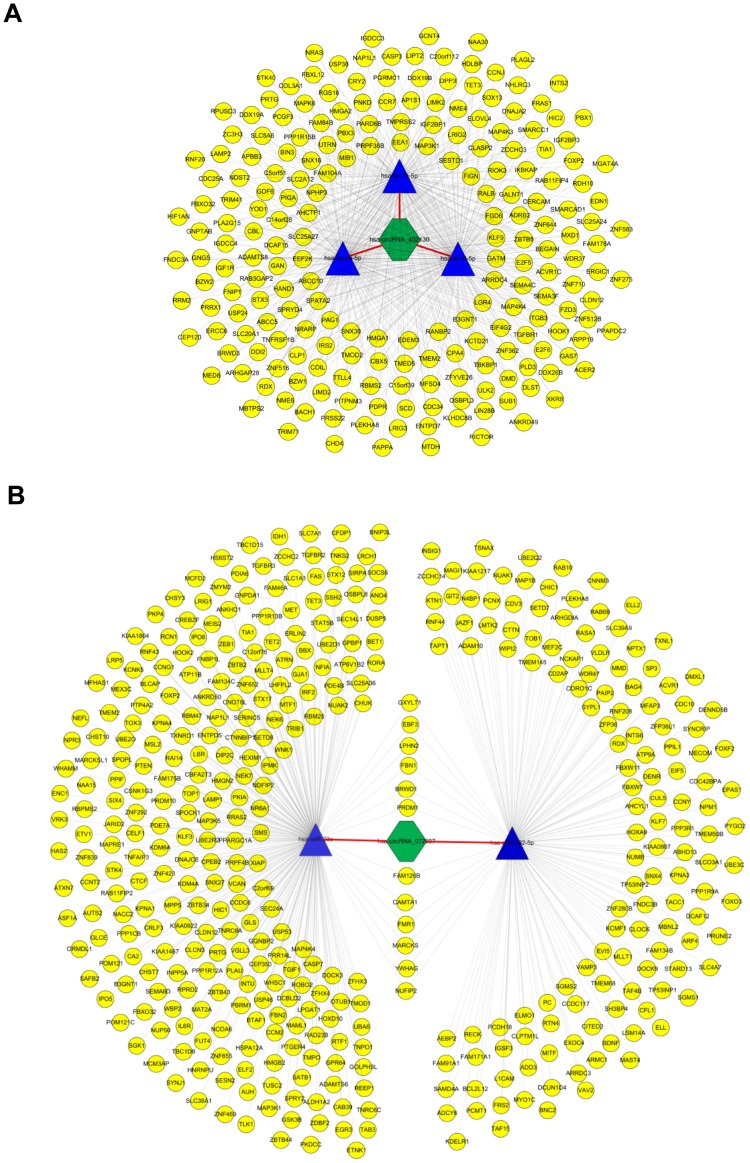
**CircRNA-miRNA-mRNA network analysis.** (**A**) The ceRNA network of hsa_circRNA_402130. The network consists of 3 miRNAs and 212 genes with 631 relationships. (**B**) The ceRNA network of hsa_circRNA_072697. The network consists of 2 miRNAs and 411 genes with 425 relationships. The yellow round node represents a protein-coding gene, blue triangle node represents a miRNA and a green hexagon represents a circRNA.

To dissect out the functions of the two validated circRNAs, starBase v2.0 and miRDB identified down-stream target genes of the abovementioned candidate miRNAs. The two circRNA-miRNA-mRNA regulatory networks (built by OmicShare tools) included 3 miRNAs and 212 mRNAs in the ceRNA network of hsa_circRNA_402130 (Figure 5A, Supplementary Table 1), and 2 miRNAs and 411 mRNAs in the ceRNA network of hsa_circRNA_072697 (Figure 5B, Supplementary Table 2). As shown in Figure 5A, we found that this network contains multiple transcription factors such as HMGA2, SOX13, E2F5, LIN28B and so on. Similarly, multiple transcription factors such as FOXO3, FOXP2 and SP3 are included in the regulatory network of Figure 5B. These regulatory networks revealed that circRNAs may play a crucial part in the process of NOA.

### GO and KEGG enrichment analysis of the predicted network genes for hsa_circRNA_402130 and hsa_circRNA_072697

**Figure 6:**
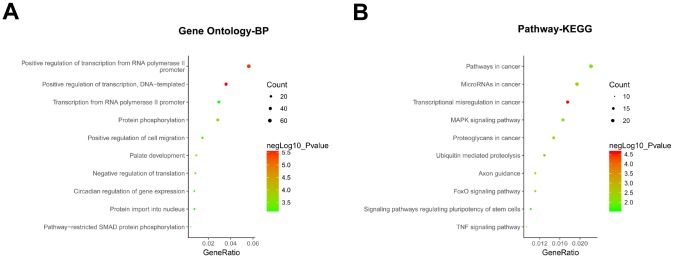
**GO and KEGG enrichment analysis of hsa_circRNA_402130 and hsa_circRNA_072697.** (**A**) Gene ontology (GO) enrichment analysis of the two circRNAs predicted target genes in terms of biological processes (BP). (**B**) KEGG enrichment analysis of the two circRNAs predicted target genes. The top 10 significantly enriched activities were shown above. The size of each circle indicates the counting number on each part, while the color represents the negative logarithm of p value of the enrichment analysis.

The functions of all the target genes were analyzed by online biological classification tool DAVID. The top 10 GO terms and KEGG terms are displayed as bubble diagram. The GO biological process (BP) analysis (Figure 6A) showed that the target genes were significantly enriched in positive regulations including transcription from RNA polymerase II promoter, transcription, cell migration, protein phosphorylation, palate development, pathway-restricted SMAD protein phosphorylation, circadian regulation of gene expression. KEGG pathway analysis (Figure 6B), showed target genes were enriched for microRNAs in cancer, MAPK signaling pathway, signaling pathways regulating pluripotency of stem cells, FoxO signaling pathway, ubiquitin mediated proteolysis, axon guidance, transcriptional misregulation in cancer, proteoglycans in cancer and TNF signaling pathway. These findings imply that circRNAs might participate in the development of NOA through multiple signaling pathways.

## DISCUSSION

Results from the Human Genome Project and the DNA Elements Encyclopedia Project indicated that protein-coding gene sequences account for only 1-3% of the human genome sequence. Also, most of the transcribed sequences in the human genome are non-coding RNA sequences [24]. Studies demonstrated that non-coding RNAs including miRNAs and lncRNAs participate in the occurrence and development of NOA [13, 18, 22, 23, 25–27]. However, the role of circRNAs, a class of non-coding RNA, has not been reported in NOA.

Here, we constructed the expression profile of circRNAs in NOA by high-throughput circRNA microarray for the first time. 16 up-regulated and 66 down-regulated circRNAs in NOA were identified. As most of these circRNAs originate from exons or introns, these results imply that dysregulated expression of circRNAs may be involved in the process of NOA. Subsequently, differential expression of circRNAs was confirmed by qRT-PCR. As circRNAs usually function as RNA sponge to regulate gene expression [4, 20, 21], bioinformatics was used to predict target miRNAs of hsa_circRNA_402130 and hsa_circRNA_072697. The results showed that hsa-let-7b-5p, hsa-let-7c-5p and hsa-let-7i-5p had a binding site for hsa_circRNA_402130, and hsa-miR-23c and hsa-miR-182-5p had a binding site for hsa_circRNA_ 072697. These indicated that hsa-let-7c-5p, hsa-let-7b-5p and hsa-let-7i-5p were the targets for hsa_ circRNA_402130, and hsa-miR-23c and hsa-miR-182-5p were the targets for hsa_circRNA_072697. In addition, these predicted miRNAs were previously reported that hsa-let-7b-5p, hsa-let-7c-5p and hsa-let-7i-5p were up-regulated and hsa-miR-23c and hsa-miR-182-5p were down-regulated in NOA [22, 23]. Among these target miRNAs, let-7 family is an important regulator of stemness by regulating LIN28A, a gene that plays pivotal roles in organogenesis, embryonic development and tumorigenesis [28–30]. Jixin Tang et al. [31] indicated that let-7 miRNA family (let-7c-5p and let-7i-5p) regulate spermatogenesis in mice through LIN28A. Another member of the let-7 miRNA family let-7g could significantly reduce the germ cell pool in mice [32]. Based on these findings, we speculate that hsa_circRNA_402130 may cause NOA by regulating the expression of let-7 miRNA family.

Moreover, based on the above two circRNAs, we constructed “circRNA-miRNA-mRNA” ceRNA regulatory networks. From these regulatory networks, we found many transcription factors that regulate spermatogenesis. Studies have shown that SP3 presents a unique expression pattern during spermatogenesis in mice and other mammalian [33, 34]. It has been confirmed that HMGA2 was expressed during the process from spermatocyte to spermatid in mice, and the phenotype of HMGA2-null mice was few spermatids and complete absence of spermatozoa [35]. We also found some protein coding genes that are affected by let-7 miRNAs involved in spermatogenesis. TET3 is expressed in round spermatids and sperm and its effect on sperm methylome might be essential in human [36]. As early as 2006, a group found that CDC25A was down-regulated in the testis tissue of male infertile patients [37]. Subsequent studies suggested that CDC25A might be regulated by BOLL and participate in human spermatogenesis [38]. These evidences all indirectly indicated that circRNAs were involved in spermatogenesis. However, experiments are required to confirm these interactions and, to elucidate the localization of circRNAs and target miRNAs.

Additionally, we also performed GO and KEGG enrichment analysis of miRNA target genes for the first time. GO enrichment analysis revealed miRNA target genes that positively regulate transcription from RNA polymerase II promoter, transcription, cell migration, protein phosphorylation, palate development, pathway-restricted SMAD protein phosphorylation, circadian regulation of gene expression and so on. Given that phosphorylation of SMAD promotes differentiation of mouse SSC [39], and proliferation of germ cells in mice [40], it is possible that circRNAs may regulate spermatogenesis by affecting SMAD activity in NOA patients. Among the KEGG pathways associated with the two circRNAs, FoxO signaling pathway and MAPK signaling pathway are strongly associated with spermatogenesis in mice [41–43]. Based on our and previous data, we can easily say that humans and mice share many common ways in the regulation of spermatogenesis. More studies on NOA patients should be conducted to improve our understanding of the mechanisms of spermatogenesis and NOA to validate the results of animal experiments. In addition, we identified a signaling pathway involved in regulation pluripotency of stem cells within the network of the dysregulated circRNAs. This is significant as self-renewal and mitosis of spermatogonial stem cells are important steps in human spermatogenesis. Based on the abovementioned ceRNA networks and enrichment analysis results, we have discovered many molecules and pathways related to stemness. Thus, we suspect that these two circRNAs may be involved in the development of NOA by regulating the stemness of spermatogonial stem cells. In a word, our study provides new ideas and strategies for NOA research. But whether circRNAs are involved in these processes deserves to be confirmed by subsequent *in vitro* and *in vivo* experiments.

Our study also has two limitations. First, more testicular tissue samples could be included in this study. This might reduce the error caused by individual differences of patients. In addition, due to the difficulties in acquiring testicular samples, we were unable to use the testicular samples of volunteers who had already been known fertility and normal spermatogenesis as normal controls.

In summary, this is the first report that reveals the expression profile of differentially expressed circRNAs and, in conjunction with bioinformatics analysis, provides the first assessment of ceRNA networks and circRNAs associated pathways in NOA. Results indicated that circRNAs may play important functions and have potential to become therapeutic targets for NOA.

## MATERIALS AND METHODS

### Testicular tissue samples collection

The study was approved by the Ethics Committee of the Institute of Human Reproduction and Stem Cell Engineering of Central South University. Testicular biopsies were collected from 6 NOA and 3 OA patients. All participants signed informed consent, and routine semen analysis based on the World Health Organization criteria showed no sperm. Most patients have been tested for follicle-stimulating hormone (FSH), luteinizing hormone (LH) and testosterone (T). All the patients underwent testicular fine needle aspiration for histological examination at Reproductive and Genetic Hospital of CITIC-Xiangya. Those patients without testicular sperm were defined as NOA patients. OA patients had normal spermatogenic function accompanied by blockage of the vas deferens, but no other congenital diseases. The results of the pathological examinations are in the (Supplementary Figure 1). None of the patients were exposed to radiation, high temperature or toxic substances prior to their hospitalization. NOA patients with chromosomal abnormalities, Y chromosome microdeletion, and varicocele were excluded from the study.

### Total RNA extraction

Nine RNA samples of testicular biopsies from NOA and OA patients were extracted using TRIzol (Invitrogen, USA) following the manufacturer’s protocol. The quantity and purity of total RNA was measured on the NanoDrop® ND-1000 spectrophotometer (Thermo Fisher, USA). RNA integrity was measured by denaturing agarose gel electrophoresis.

### Microarray detection

All RNA samples were analyzed by Arraystar circRNA Microarray at Kangchen Bio-tech (Shanghai, China). Sample labeling and array hybridization were performed according to the manufacturer’s protocol (Arraystar Inc.). The hybridized arrays were washed, fixed and scanned on the Agilent Scanner G2505C. Agilent Feature Extraction software was used for raw data extraction. The data was then processed using the limma package of R software. Low intensity was filtered after quantile normalization of the raw data. Student’s t-test was used to estimate the statistical significance between groups. CircRNAs with a fold change ≥ 1.5 and p < 0.05 were considered significant.

### Quantitative real-time reverse transcription PCR

The cDNA was prepared from 1ug of total RNA using Super Script TMIII Reverse Transcriptase kit (Invitrogen) according to the manufacturer’s instructions. Quantitative real-time reverse transcription PCR (qRT-PCR) was performed using a 2×PCR master mix (Arraystar) on the ViiA 7 Real-time PCR System (Applied Biosystems) to detect the relative expression levels of target circRNAs. The primer sequences for the target circRNAs are as follows: hsa_ circRNA_402130 forward: 5′-GTGGCCGAGGACTTTGATTG-3′, reverse: 5′-CCTGTAACAACGCATCTCATATT-3′; hsa_circRNA_100812 forward: 5′-TATTCTCAAGCTGTCACAGGACATT-3′, reverse: 5′-TGAGGGTAGCAGCAGAACGAG-3′; hsa_circRNA_072697 forward: 5′-TGATAGAAAAGTTAGAATTTTCAGA-3′, reverse: 5′-ACTCTTTCAAACTCTAAGAGCTTAG-3′; hsa_circRNA_104078 forward: 5′-GCTTATGGCTATAAAATTACAGAGA-3′, reverse: 5′-CGGGACAACATCCTTTCTTAC-3′; hsa_circRNA_030050 forward: 5′-GGGAGAAGCAGCTAGAACCA-3′; reverse: 5′-TT TGCCAGAATACCCCTTTG-3′; hsa_circRNA_406168 forward: 5′-ATTGGGTTCTTTGCCTGTTG-3′; reverse: 5′-GGGGCAGACAGATGAGAAAG-3′; β-actin forward: 5′-GTGGCCGAGGACTTTGATTG-3′, reverse: 5′-CCTGTAACAACGCATCTCATATT-3′. Transcript level of the housekeeping gene β-actin was used to normalize the relative expression of circRNAs. Data is represented as mean ± SE of three independent experiments.

### Annotation and functional prediction of hsa_circRNA_402130 and hsa_circRNA_072697

The circRNA-microRNA interactions of hsa_circRNA_402130 and hsa_circRNA_072697 were predicted with Arraystar’s home-made microRNA target prediction software based on TargetScan [44] and miRanda [45]. The analysis process of the software is as follows: First, it performs target prediction by using the miRNA information in the latest miRBase on the target sequence. Then, candidate miRNAs are screened by Context+<=-0.05 and Context<=-0.05. Finally, according to the miRNA coverage of MREs (MicroRNA Recoginition Elements) on the target sequence, the required circRNA-microRNA interactions are obtained. The microRNA-mRNA interactions were predicted by starBase v2.0 (http://starbase.sysu.edu.cn/browser.php) [46, 47] and miRDB (http://www.mirdb.org) [48, 49]. GO and KEGG enrichment analysis of circRNA-targeting genes were performed by DAVID https://david.ncifcrf.gov/. Then OmicShare tools (http://www.omicshare.com/tools) was used to create potential maps of the circRNA/miRNA/mRNA interaction networks of hsa_circ_402130 and hsa_circ_072697.

### Statistical analysis

Student’s t-test (two-tailed) was used to estimate statistical significance between groups. Data analysis was performed using GraphPad Prism 5.0 (GraphPad Software, La Jolla, CA). A p-value < 0.05 was considered significant.

## Supplementary Material

Supplementary Figure 1

Supplementary Table 1

Supplementary Table 2
