# Supplementary material for: Testicular biopsies microarray analysis reveals circRNAs are involved in the pathogenesis of non-obstructive azoospermia
Source: Aging (Albany NY). 2020 Feb 6;12(3):2610–25. doi: 10.18632/aging.102765 (PMC7041731; doi:10.18632/aging.102765)
Supplement: Supplementary Figure 1 [file aging-12-102765-s003..pdf]

SUPPLEMENTARY FIGURE

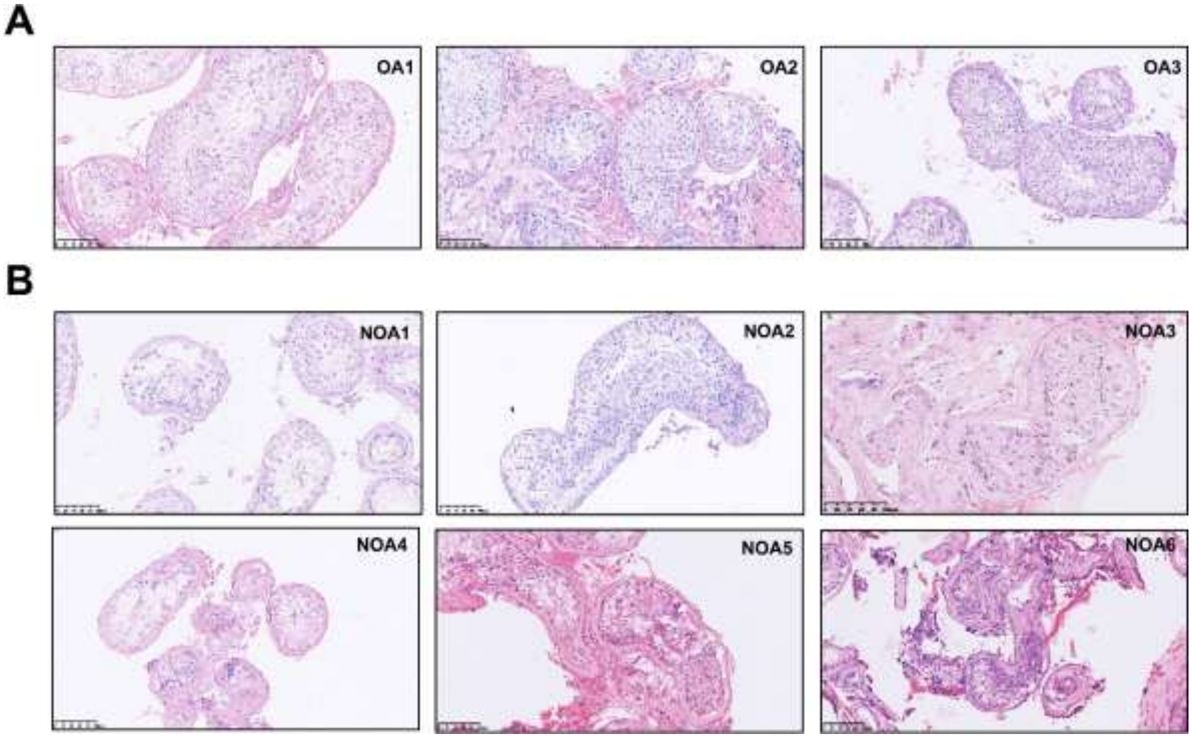

Supplementary Figure 1. Histological pathological examinations of testis samples from 3 OA and 10 NOA patients. OA: obstructive azoospermia; NOA: non-obstructive azoospermia. Scale bar=100 μm.
