# Supplementary material for: Testicular biopsies microarray analysis reveals circRNAs are involved in the pathogenesis of non-obstructive azoospermia
Source: Aging (Albany NY). 2020 Feb 6;12(3):2610–25. doi: 10.18632/aging.102765 (PMC7041731; doi:10.18632/aging.102765)
Supplement: Supplementary Table 1 [file aging-12-102765-s001..doc]

Supplementary Table 1.

| **miRNA** | **target** |
| --- | --- |
| hsa-let-7b-5p | hsa-circRNA_402130 |
| hsa-let-7c-5p | hsa-circRNA_402130 |
| hsa-let-7i-5p | hsa-circRNA_402130 |
| hsa-let-7b-5p | ERGIC1 |
| hsa-let-7b-5p | CLDN12 |
| hsa-let-7b-5p | STX3 |
| hsa-let-7b-5p | DDX19A |
| hsa-let-7b-5p | EDN1 |
| hsa-let-7b-5p | COL3A1 |
| hsa-let-7b-5p | PRTG |
| hsa-let-7b-5p | FAM178A |
| hsa-let-7b-5p | NHLRC3 |
| hsa-let-7b-5p | FBXL12 |
| hsa-let-7b-5p | FRAS1 |
| hsa-let-7b-5p | RDH10 |
| hsa-let-7b-5p | HIC2 |
| hsa-let-7b-5p | FOXP2 |
| hsa-let-7b-5p | IGF2BP3 |
| hsa-let-7b-5p | TIA1 |
| hsa-let-7b-5p | SMARCC1 |
| hsa-let-7b-5p | DPP3 |
| hsa-let-7b-5p | TET3 |
| hsa-let-7b-5p | SOX13 |
| hsa-let-7b-5p | DNAJA2 |
| hsa-let-7b-5p | PGRMC1 |
| hsa-let-7b-5p | DDX19B |
| hsa-let-7b-5p | RGS16 |
| hsa-let-7b-5p | CRY2 |
| hsa-let-7b-5p | PCGF3 |
| hsa-let-7b-5p | MAPK6 |
| hsa-let-7b-5p | UTRN |
| hsa-let-7b-5p | SLC5A6 |
| hsa-let-7b-5p | SNX16 |
| hsa-let-7b-5p | PPP1R15B |
| hsa-let-7b-5p | APBB3 |
| hsa-let-7b-5p | SLC2A12 |
| hsa-let-7b-5p | BIN3 |
| hsa-let-7b-5p | C5orf51 |
| hsa-let-7b-5p | YOD1 |
| hsa-let-7b-5p | CCNJ |
| hsa-let-7b-5p | PIGA |
| hsa-let-7b-5p | GDF6 |
| hsa-let-7b-5p | CBL |
| hsa-let-7b-5p | NDST2 |
| hsa-let-7b-5p | C14orf28 |
| hsa-let-7b-5p | DCAF15 |
| hsa-let-7b-5p | GAN |
| hsa-let-7b-5p | NRARP |
| hsa-let-7b-5p | HAND1 |
| hsa-let-7b-5p | ADAMTS8 |
| hsa-let-7b-5p | TRIM41 |
| hsa-let-7b-5p | RAB3GAP2 |
| hsa-let-7b-5p | PLA2G15 |
| hsa-let-7b-5p | ZC3H3 |
| hsa-let-7b-5p | SPRYD4 |
| hsa-let-7b-5p | FNIP1 |
| hsa-let-7b-5p | IGDCC4 |
| hsa-let-7b-5p | GNG5 |
| hsa-let-7b-5p | SLC25A27 |
| hsa-let-7b-5p | USP24 |
| hsa-let-7b-5p | GNPTAB |
| hsa-let-7b-5p | ABCC10 |
| hsa-let-7b-5p | FNDC3A |
| hsa-let-7b-5p | RRM2 |
| hsa-let-7b-5p | PAG1 |
| hsa-let-7b-5p | SLC20A1 |
| hsa-let-7b-5p | IGF1R |
| hsa-let-7b-5p | CEP120 |
| hsa-let-7b-5p | RICTOR |
| hsa-let-7b-5p | SNX30 |
| hsa-let-7b-5p | MED8 |
| hsa-let-7b-5p | ABCC5 |
| hsa-let-7b-5p | TNFRSF1B |
| hsa-let-7b-5p | CLP1 |
| hsa-let-7b-5p | COIL |
| hsa-let-7b-5p | TTLL4 |
| hsa-let-7b-5p | PLAGL2 |
| hsa-let-7b-5p | ANKRD49 |
| hsa-let-7b-5p | C15orf39 |
| hsa-let-7b-5p | MTDH |
| hsa-let-7b-5p | MBTPS2 |
| hsa-let-7b-5p | MFSD4 |
| hsa-let-7b-5p | PAPPA |
| hsa-let-7b-5p | CHD4 |
| hsa-let-7b-5p | TRIM71 |
| hsa-let-7b-5p | RBMS2 |
| hsa-let-7b-5p | RPUSD3 |
| hsa-let-7b-5p | STK40 |
| hsa-let-7b-5p | IGDCC3 |
| hsa-let-7b-5p | ZFYVE26 |
| hsa-let-7b-5p | NRAS |
| hsa-let-7b-5p | BZW2 |
| hsa-let-7b-5p | HIF1AN |
| hsa-let-7b-5p | PBX1 |
| hsa-let-7b-5p | MGAT4A |
| hsa-let-7b-5p | INTS2 |
| hsa-let-7b-5p | RNF20 |
| hsa-let-7b-5p | ZNF583 |
| hsa-let-7b-5p | ERCC6 |
| hsa-let-7b-5p | NAA30 |
| hsa-let-7b-5p | ZNF275 |
| hsa-let-7b-5p | GCNT4 |
| hsa-let-7b-5p | PRRX1 |
| hsa-let-7b-5p | PPAPDC2 |
| hsa-let-7b-5p | ACER2 |
| hsa-let-7b-5p | ARHGAP28 |
| hsa-let-7b-5p | XKR8 |
| hsa-let-7b-5p | BACH1 |
| hsa-let-7b-5p | BRWD3 |
| hsa-let-7b-5p | ENTPD7 |
| hsa-let-7b-5p | PRSS22 |
| hsa-let-7b-5p | NME6 |
| hsa-let-7b-5p | LRIG3 |
| hsa-let-7b-5p | RDX |
| hsa-let-7b-5p | LIN28B |
| hsa-let-7b-5p | PLEKHA8 |
| hsa-let-7b-5p | SUB1 |
| hsa-let-7b-5p | KLHDC8B |
| hsa-let-7b-5p | DDX26B |
| hsa-let-7b-5p | DLST |
| hsa-let-7b-5p | GAS7 |
| hsa-let-7b-5p | ZNF512B |
| hsa-let-7b-5p | ARPP19 |
| hsa-let-7b-5p | ZNF516 |
| hsa-let-7b-5p | DDI2 |
| hsa-let-7b-5p | BZW1 |
| hsa-let-7b-5p | LIMD2 |
| hsa-let-7b-5p | PDPR |
| hsa-let-7b-5p | PITPNM3 |
| hsa-let-7b-5p | ULK2 |
| hsa-let-7b-5p | SCD |
| hsa-let-7b-5p | OSBPL3 |
| hsa-let-7b-5p | CDC34 |
| hsa-let-7b-5p | PLD3 |
| hsa-let-7b-5p | DMD |
| hsa-let-7b-5p | HOOK1 |
| hsa-let-7b-5p | E2F6 |
| hsa-let-7b-5p | ZNF710 |
| hsa-let-7b-5p | FZD3 |
| hsa-let-7b-5p | MXD1 |
| hsa-let-7b-5p | WDR37 |
| hsa-let-7b-5p | RAB11FIP4 |
| hsa-let-7b-5p | SMARCAD1 |
| hsa-let-7b-5p | MAP4K3 |
| hsa-let-7b-5p | TBKBP1 |
| hsa-let-7b-5p | ZNF362 |
| hsa-let-7b-5p | TGFBR1 |
| hsa-let-7b-5p | SEMA3F |
| hsa-let-7b-5p | ITGB3 |
| hsa-let-7b-5p | ACVR1C |
| hsa-let-7b-5p | BEGAIN |
| hsa-let-7b-5p | ZNF644 |
| hsa-let-7b-5p | CERCAM |
| hsa-let-7b-5p | ZCCHC3 |
| hsa-let-7b-5p | TMED5 |
| hsa-let-7b-5p | CBX5 |
| hsa-let-7b-5p | TMOD2 |
| hsa-let-7b-5p | IRS2 |
| hsa-let-7b-5p | TMEM2 |
| hsa-let-7b-5p | CPA4 |
| hsa-let-7b-5p | KCTD21 |
| hsa-let-7b-5p | EIF4G2 |
| hsa-let-7b-5p | MAP4K4 |
| hsa-let-7b-5p | E2F5 |
| hsa-let-7b-5p | ZBTB5 |
| hsa-let-7b-5p | ADRB2 |
| hsa-let-7b-5p | GALNT1 |
| hsa-let-7b-5p | RIOK3 |
| hsa-let-7b-5p | AHCTF1 |
| hsa-let-7b-5p | EEF2K |
| hsa-let-7b-5p | HMGA1 |
| hsa-let-7b-5p | SPATA2 |
| hsa-let-7b-5p | PRPF38B |
| hsa-let-7b-5p | RANBP2 |
| hsa-let-7b-5p | B3GNT1 |
| hsa-let-7b-5p | LGR4 |
| hsa-let-7b-5p | FBXO32 |
| hsa-let-7b-5p | ARRDC4 |
| hsa-let-7b-5p | GATM |
| hsa-let-7b-5p | KLF9 |
| hsa-let-7b-5p | FIGN |
| hsa-let-7b-5p | SESTD1 |
| hsa-let-7b-5p | MAP3K1 |
| hsa-let-7b-5p | FGD6 |
| hsa-let-7b-5p | RALB |
| hsa-let-7b-5p | EEA1 |
| hsa-let-7b-5p | EDEM3 |
| hsa-let-7b-5p | MIB1 |
| hsa-let-7b-5p | FAM104A |
| hsa-let-7b-5p | NPHP3 |
| hsa-let-7b-5p | CLASP2 |
| hsa-let-7b-5p | LRIG2 |
| hsa-let-7b-5p | SEMA4C |
| hsa-let-7b-5p | TMPRSS2 |
| hsa-let-7b-5p | PARD6B |
| hsa-let-7b-5p | PBX3 |
| hsa-let-7b-5p | IKBKAP |
| hsa-let-7b-5p | ELOVL4 |
| hsa-let-7b-5p | NME4 |
| hsa-let-7b-5p | AP1S1 |
| hsa-let-7b-5p | CCR7 |
| hsa-let-7b-5p | IGF2BP1 |
| hsa-let-7b-5p | LIMK2 |
| hsa-let-7b-5p | HMGA2 |
| hsa-let-7b-5p | SLC25A24 |
| hsa-let-7b-5p | C20orf112 |
| hsa-let-7b-5p | PNKD |
| hsa-let-7b-5p | FAM84B |
| hsa-let-7b-5p | HDLBP |
| hsa-let-7b-5p | CASP3 |
| hsa-let-7b-5p | NAP1L1 |
| hsa-let-7b-5p | LIPT2 |
| hsa-let-7b-5p | USP38 |
| hsa-let-7b-5p | CDC25A |
| hsa-let-7c-5p | ERGIC1 |
| hsa-let-7c-5p | CLDN12 |
| hsa-let-7c-5p | STX3 |
| hsa-let-7c-5p | DDX19A |
| hsa-let-7c-5p | EDN1 |
| hsa-let-7c-5p | COL3A1 |
| hsa-let-7c-5p | PRTG |
| hsa-let-7c-5p | FAM178A |
| hsa-let-7c-5p | NHLRC3 |
| hsa-let-7c-5p | FBXL12 |
| hsa-let-7c-5p | FRAS1 |
| hsa-let-7c-5p | RDH10 |
| hsa-let-7c-5p | HIC2 |
| hsa-let-7c-5p | FOXP2 |
| hsa-let-7c-5p | IGF2BP3 |
| hsa-let-7c-5p | TIA1 |
| hsa-let-7c-5p | SMARCC1 |
| hsa-let-7c-5p | DPP3 |
| hsa-let-7c-5p | TET3 |
| hsa-let-7c-5p | SOX13 |
| hsa-let-7c-5p | DNAJA2 |
| hsa-let-7c-5p | PGRMC1 |
| hsa-let-7c-5p | DDX19B |
| hsa-let-7c-5p | RGS16 |
| hsa-let-7c-5p | CRY2 |
| hsa-let-7c-5p | PCGF3 |
| hsa-let-7c-5p | MAPK6 |
| hsa-let-7c-5p | UTRN |
| hsa-let-7c-5p | SLC5A6 |
| hsa-let-7c-5p | SNX16 |
| hsa-let-7c-5p | PPP1R15B |
| hsa-let-7c-5p | APBB3 |
| hsa-let-7c-5p | SLC2A12 |
| hsa-let-7c-5p | BIN3 |
| hsa-let-7c-5p | C5orf51 |
| hsa-let-7c-5p | YOD1 |
| hsa-let-7c-5p | CCNJ |
| hsa-let-7c-5p | PIGA |
| hsa-let-7c-5p | GDF6 |
| hsa-let-7c-5p | CBL |
| hsa-let-7c-5p | NDST2 |
| hsa-let-7c-5p | C14orf28 |
| hsa-let-7c-5p | DCAF15 |
| hsa-let-7c-5p | GAN |
| hsa-let-7c-5p | NRARP |
| hsa-let-7c-5p | HAND1 |
| hsa-let-7c-5p | ADAMTS8 |
| hsa-let-7c-5p | TRIM41 |
| hsa-let-7c-5p | RAB3GAP2 |
| hsa-let-7c-5p | PLA2G15 |
| hsa-let-7c-5p | ZC3H3 |
| hsa-let-7c-5p | SPRYD4 |
| hsa-let-7c-5p | FNIP1 |
| hsa-let-7c-5p | IGDCC4 |
| hsa-let-7c-5p | GNG5 |
| hsa-let-7c-5p | SLC25A27 |
| hsa-let-7c-5p | USP24 |
| hsa-let-7c-5p | GNPTAB |
| hsa-let-7c-5p | ABCC10 |
| hsa-let-7c-5p | FNDC3A |
| hsa-let-7c-5p | RRM2 |
| hsa-let-7c-5p | PAG1 |
| hsa-let-7c-5p | SLC20A1 |
| hsa-let-7c-5p | IGF1R |
| hsa-let-7c-5p | CEP120 |
| hsa-let-7c-5p | RICTOR |
| hsa-let-7c-5p | SNX30 |
| hsa-let-7c-5p | MED8 |
| hsa-let-7c-5p | ABCC5 |
| hsa-let-7c-5p | TNFRSF1B |
| hsa-let-7c-5p | CLP1 |
| hsa-let-7c-5p | COIL |
| hsa-let-7c-5p | TTLL4 |
| hsa-let-7c-5p | PLAGL2 |
| hsa-let-7c-5p | ANKRD49 |
| hsa-let-7c-5p | C15orf39 |
| hsa-let-7c-5p | MTDH |
| hsa-let-7c-5p | MBTPS2 |
| hsa-let-7c-5p | MFSD4 |
| hsa-let-7c-5p | PAPPA |
| hsa-let-7c-5p | CHD4 |
| hsa-let-7c-5p | TRIM71 |
| hsa-let-7c-5p | RBMS2 |
| hsa-let-7c-5p | RPUSD3 |
| hsa-let-7c-5p | STK40 |
| hsa-let-7c-5p | IGDCC3 |
| hsa-let-7c-5p | ZFYVE26 |
| hsa-let-7c-5p | NRAS |
| hsa-let-7c-5p | BZW2 |
| hsa-let-7c-5p | HIF1AN |
| hsa-let-7c-5p | PBX1 |
| hsa-let-7c-5p | MGAT4A |
| hsa-let-7c-5p | INTS2 |
| hsa-let-7c-5p | RNF20 |
| hsa-let-7c-5p | ZNF583 |
| hsa-let-7c-5p | ERCC6 |
| hsa-let-7c-5p | NAA30 |
| hsa-let-7c-5p | ZNF275 |
| hsa-let-7c-5p | GCNT4 |
| hsa-let-7c-5p | PRRX1 |
| hsa-let-7c-5p | PPAPDC2 |
| hsa-let-7c-5p | ACER2 |
| hsa-let-7c-5p | ARHGAP28 |
| hsa-let-7c-5p | XKR8 |
| hsa-let-7c-5p | BACH1 |
| hsa-let-7c-5p | BRWD3 |
| hsa-let-7c-5p | ENTPD7 |
| hsa-let-7c-5p | PRSS22 |
| hsa-let-7c-5p | NME6 |
| hsa-let-7c-5p | LRIG3 |
| hsa-let-7c-5p | RDX |
| hsa-let-7c-5p | LIN28B |
| hsa-let-7c-5p | PLEKHA8 |
| hsa-let-7c-5p | SUB1 |
| hsa-let-7c-5p | KLHDC8B |
| hsa-let-7c-5p | DDX26B |
| hsa-let-7c-5p | DLST |
| hsa-let-7c-5p | GAS7 |
| hsa-let-7c-5p | ZNF512B |
| hsa-let-7c-5p | ARPP19 |
| hsa-let-7c-5p | ZNF516 |
| hsa-let-7c-5p | DDI2 |
| hsa-let-7c-5p | BZW1 |
| hsa-let-7c-5p | LIMD2 |
| hsa-let-7c-5p | PDPR |
| hsa-let-7c-5p | PITPNM3 |
| hsa-let-7c-5p | ULK2 |
| hsa-let-7c-5p | SCD |
| hsa-let-7c-5p | OSBPL3 |
| hsa-let-7c-5p | CDC34 |
| hsa-let-7c-5p | PLD3 |
| hsa-let-7c-5p | DMD |
| hsa-let-7c-5p | HOOK1 |
| hsa-let-7c-5p | E2F6 |
| hsa-let-7c-5p | ZNF710 |
| hsa-let-7c-5p | FZD3 |
| hsa-let-7c-5p | MXD1 |
| hsa-let-7c-5p | WDR37 |
| hsa-let-7c-5p | RAB11FIP4 |
| hsa-let-7c-5p | SMARCAD1 |
| hsa-let-7c-5p | MAP4K3 |
| hsa-let-7c-5p | TBKBP1 |
| hsa-let-7c-5p | ZNF362 |
| hsa-let-7c-5p | TGFBR1 |
| hsa-let-7c-5p | SEMA3F |
| hsa-let-7c-5p | ITGB3 |
| hsa-let-7c-5p | ACVR1C |
| hsa-let-7c-5p | BEGAIN |
| hsa-let-7c-5p | ZNF644 |
| hsa-let-7c-5p | CERCAM |
| hsa-let-7c-5p | ZCCHC3 |
| hsa-let-7c-5p | TMED5 |
| hsa-let-7c-5p | CBX5 |
| hsa-let-7c-5p | TMOD2 |
| hsa-let-7c-5p | IRS2 |
| hsa-let-7c-5p | TMEM2 |
| hsa-let-7c-5p | CPA4 |
| hsa-let-7c-5p | KCTD21 |
| hsa-let-7c-5p | EIF4G2 |
| hsa-let-7c-5p | MAP4K4 |
| hsa-let-7c-5p | E2F5 |
| hsa-let-7c-5p | ZBTB5 |
| hsa-let-7c-5p | ADRB2 |
| hsa-let-7c-5p | GALNT1 |
| hsa-let-7c-5p | RIOK3 |
| hsa-let-7c-5p | AHCTF1 |
| hsa-let-7c-5p | EEF2K |
| hsa-let-7c-5p | HMGA1 |
| hsa-let-7c-5p | SPATA2 |
| hsa-let-7c-5p | PRPF38B |
| hsa-let-7c-5p | RANBP2 |
| hsa-let-7c-5p | B3GNT1 |
| hsa-let-7c-5p | LGR4 |
| hsa-let-7c-5p | ARRDC4 |
| hsa-let-7c-5p | GATM |
| hsa-let-7c-5p | KLF9 |
| hsa-let-7c-5p | FIGN |
| hsa-let-7c-5p | SESTD1 |
| hsa-let-7c-5p | MAP3K1 |
| hsa-let-7c-5p | FGD6 |
| hsa-let-7c-5p | RALB |
| hsa-let-7c-5p | EEA1 |
| hsa-let-7c-5p | EDEM3 |
| hsa-let-7c-5p | MIB1 |
| hsa-let-7c-5p | FAM104A |
| hsa-let-7c-5p | NPHP3 |
| hsa-let-7c-5p | CLASP2 |
| hsa-let-7c-5p | LRIG2 |
| hsa-let-7c-5p | SEMA4C |
| hsa-let-7c-5p | TMPRSS2 |
| hsa-let-7c-5p | PARD6B |
| hsa-let-7c-5p | PBX3 |
| hsa-let-7c-5p | IKBKAP |
| hsa-let-7c-5p | ELOVL4 |
| hsa-let-7c-5p | NME4 |
| hsa-let-7c-5p | AP1S1 |
| hsa-let-7c-5p | CCR7 |
| hsa-let-7c-5p | IGF2BP1 |
| hsa-let-7c-5p | LIMK2 |
| hsa-let-7c-5p | HMGA2 |
| hsa-let-7c-5p | SLC25A24 |
| hsa-let-7c-5p | C20orf112 |
| hsa-let-7c-5p | PNKD |
| hsa-let-7c-5p | FAM84B |
| hsa-let-7c-5p | HDLBP |
| hsa-let-7c-5p | CASP3 |
| hsa-let-7c-5p | NAP1L1 |
| hsa-let-7c-5p | LIPT2 |
| hsa-let-7c-5p | USP38 |
| hsa-let-7c-5p | CDC25A |
| hsa-let-7i-5p | ERGIC1 |
| hsa-let-7i-5p | CLDN12 |
| hsa-let-7i-5p | STX3 |
| hsa-let-7i-5p | DDX19A |
| hsa-let-7i-5p | EDN1 |
| hsa-let-7i-5p | COL3A1 |
| hsa-let-7i-5p | PRTG |
| hsa-let-7i-5p | FAM178A |
| hsa-let-7i-5p | NHLRC3 |
| hsa-let-7i-5p | FBXL12 |
| hsa-let-7i-5p | FRAS1 |
| hsa-let-7i-5p | RDH10 |
| hsa-let-7i-5p | HIC2 |
| hsa-let-7i-5p | FOXP2 |
| hsa-let-7i-5p | IGF2BP3 |
| hsa-let-7i-5p | TIA1 |
| hsa-let-7i-5p | SMARCC1 |
| hsa-let-7i-5p | DPP3 |
| hsa-let-7i-5p | TET3 |
| hsa-let-7i-5p | SOX13 |
| hsa-let-7i-5p | DNAJA2 |
| hsa-let-7i-5p | PGRMC1 |
| hsa-let-7i-5p | DDX19B |
| hsa-let-7i-5p | RGS16 |
| hsa-let-7i-5p | CRY2 |
| hsa-let-7i-5p | PCGF3 |
| hsa-let-7i-5p | MAPK6 |
| hsa-let-7i-5p | UTRN |
| hsa-let-7i-5p | SLC5A6 |
| hsa-let-7i-5p | SNX16 |
| hsa-let-7i-5p | PPP1R15B |
| hsa-let-7i-5p | APBB3 |
| hsa-let-7i-5p | SLC2A12 |
| hsa-let-7i-5p | BIN3 |
| hsa-let-7i-5p | C5orf51 |
| hsa-let-7i-5p | YOD1 |
| hsa-let-7i-5p | CCNJ |
| hsa-let-7i-5p | PIGA |
| hsa-let-7i-5p | GDF6 |
| hsa-let-7i-5p | CBL |
| hsa-let-7i-5p | NDST2 |
| hsa-let-7i-5p | C14orf28 |
| hsa-let-7i-5p | DCAF15 |
| hsa-let-7i-5p | GAN |
| hsa-let-7i-5p | NRARP |
| hsa-let-7i-5p | HAND1 |
| hsa-let-7i-5p | ADAMTS8 |
| hsa-let-7i-5p | TRIM41 |
| hsa-let-7i-5p | RAB3GAP2 |
| hsa-let-7i-5p | PLA2G15 |
| hsa-let-7i-5p | ZC3H3 |
| hsa-let-7i-5p | SPRYD4 |
| hsa-let-7i-5p | FNIP1 |
| hsa-let-7i-5p | IGDCC4 |
| hsa-let-7i-5p | GNG5 |
| hsa-let-7i-5p | SLC25A27 |
| hsa-let-7i-5p | USP24 |
| hsa-let-7i-5p | GNPTAB |
| hsa-let-7i-5p | ABCC10 |
| hsa-let-7i-5p | FNDC3A |
| hsa-let-7i-5p | RRM2 |
| hsa-let-7i-5p | PAG1 |
| hsa-let-7i-5p | SLC20A1 |
| hsa-let-7i-5p | IGF1R |
| hsa-let-7i-5p | CEP120 |
| hsa-let-7i-5p | RICTOR |
| hsa-let-7i-5p | SNX30 |
| hsa-let-7i-5p | MED8 |
| hsa-let-7i-5p | ABCC5 |
| hsa-let-7i-5p | TNFRSF1B |
| hsa-let-7i-5p | CLP1 |
| hsa-let-7i-5p | COIL |
| hsa-let-7i-5p | TTLL4 |
| hsa-let-7i-5p | PLAGL2 |
| hsa-let-7i-5p | ANKRD49 |
| hsa-let-7i-5p | C15orf39 |
| hsa-let-7i-5p | MTDH |
| hsa-let-7i-5p | MBTPS2 |
| hsa-let-7i-5p | MFSD4 |
| hsa-let-7i-5p | PAPPA |
| hsa-let-7i-5p | CHD4 |
| hsa-let-7i-5p | TRIM71 |
| hsa-let-7i-5p | RBMS2 |
| hsa-let-7i-5p | RPUSD3 |
| hsa-let-7i-5p | STK40 |
| hsa-let-7i-5p | IGDCC3 |
| hsa-let-7i-5p | ZFYVE26 |
| hsa-let-7i-5p | NRAS |
| hsa-let-7i-5p | BZW2 |
| hsa-let-7i-5p | HIF1AN |
| hsa-let-7i-5p | PBX1 |
| hsa-let-7i-5p | MGAT4A |
| hsa-let-7i-5p | INTS2 |
| hsa-let-7i-5p | RNF20 |
| hsa-let-7i-5p | ZNF583 |
| hsa-let-7i-5p | ERCC6 |
| hsa-let-7i-5p | NAA30 |
| hsa-let-7i-5p | ZNF275 |
| hsa-let-7i-5p | GCNT4 |
| hsa-let-7i-5p | PRRX1 |
| hsa-let-7i-5p | PPAPDC2 |
| hsa-let-7i-5p | ACER2 |
| hsa-let-7i-5p | ARHGAP28 |
| hsa-let-7i-5p | XKR8 |
| hsa-let-7i-5p | BACH1 |
| hsa-let-7i-5p | BRWD3 |
| hsa-let-7i-5p | ENTPD7 |
| hsa-let-7i-5p | NME6 |
| hsa-let-7i-5p | LRIG3 |
| hsa-let-7i-5p | RDX |
| hsa-let-7i-5p | LIN28B |
| hsa-let-7i-5p | PLEKHA8 |
| hsa-let-7i-5p | SUB1 |
| hsa-let-7i-5p | KLHDC8B |
| hsa-let-7i-5p | DDX26B |
| hsa-let-7i-5p | DLST |
| hsa-let-7i-5p | GAS7 |
| hsa-let-7i-5p | ZNF512B |
| hsa-let-7i-5p | ARPP19 |
| hsa-let-7i-5p | ZNF516 |
| hsa-let-7i-5p | LAMP2 |
| hsa-let-7i-5p | DDI2 |
| hsa-let-7i-5p | BZW1 |
| hsa-let-7i-5p | LIMD2 |
| hsa-let-7i-5p | PDPR |
| hsa-let-7i-5p | PITPNM3 |
| hsa-let-7i-5p | ULK2 |
| hsa-let-7i-5p | SCD |
| hsa-let-7i-5p | OSBPL3 |
| hsa-let-7i-5p | CDC34 |
| hsa-let-7i-5p | PLD3 |
| hsa-let-7i-5p | DMD |
| hsa-let-7i-5p | HOOK1 |
| hsa-let-7i-5p | E2F6 |
| hsa-let-7i-5p | ZNF710 |
| hsa-let-7i-5p | FZD3 |
| hsa-let-7i-5p | MXD1 |
| hsa-let-7i-5p | WDR37 |
| hsa-let-7i-5p | RAB11FIP4 |
| hsa-let-7i-5p | SMARCAD1 |
| hsa-let-7i-5p | MAP4K3 |
| hsa-let-7i-5p | TBKBP1 |
| hsa-let-7i-5p | ZNF362 |
| hsa-let-7i-5p | TGFBR1 |
| hsa-let-7i-5p | SEMA3F |
| hsa-let-7i-5p | ITGB3 |
| hsa-let-7i-5p | ACVR1C |
| hsa-let-7i-5p | BEGAIN |
| hsa-let-7i-5p | ZNF644 |
| hsa-let-7i-5p | CERCAM |
| hsa-let-7i-5p | ZCCHC3 |
| hsa-let-7i-5p | TMED5 |
| hsa-let-7i-5p | CBX5 |
| hsa-let-7i-5p | TMOD2 |
| hsa-let-7i-5p | IRS2 |
| hsa-let-7i-5p | TMEM2 |
| hsa-let-7i-5p | CPA4 |
| hsa-let-7i-5p | KCTD21 |
| hsa-let-7i-5p | EIF4G2 |
| hsa-let-7i-5p | MAP4K4 |
| hsa-let-7i-5p | E2F5 |
| hsa-let-7i-5p | ZBTB5 |
| hsa-let-7i-5p | ADRB2 |
| hsa-let-7i-5p | GALNT1 |
| hsa-let-7i-5p | RIOK3 |
| hsa-let-7i-5p | AHCTF1 |
| hsa-let-7i-5p | EEF2K |
| hsa-let-7i-5p | HMGA1 |
| hsa-let-7i-5p | SPATA2 |
| hsa-let-7i-5p | PRPF38B |
| hsa-let-7i-5p | RANBP2 |
| hsa-let-7i-5p | B3GNT1 |
| hsa-let-7i-5p | LGR4 |
| hsa-let-7i-5p | FBXO32 |
| hsa-let-7i-5p | ARRDC4 |
| hsa-let-7i-5p | GATM |
| hsa-let-7i-5p | KLF9 |
| hsa-let-7i-5p | FIGN |
| hsa-let-7i-5p | SESTD1 |
| hsa-let-7i-5p | MAP3K1 |
| hsa-let-7i-5p | FGD6 |
| hsa-let-7i-5p | RALB |
| hsa-let-7i-5p | EEA1 |
| hsa-let-7i-5p | EDEM3 |
| hsa-let-7i-5p | MIB1 |
| hsa-let-7i-5p | FAM104A |
| hsa-let-7i-5p | NPHP3 |
| hsa-let-7i-5p | CLASP2 |
| hsa-let-7i-5p | LRIG2 |
| hsa-let-7i-5p | SEMA4C |
| hsa-let-7i-5p | TMPRSS2 |
| hsa-let-7i-5p | PARD6B |
| hsa-let-7i-5p | PBX3 |
| hsa-let-7i-5p | IKBKAP |
| hsa-let-7i-5p | ELOVL4 |
| hsa-let-7i-5p | NME4 |
| hsa-let-7i-5p | AP1S1 |
| hsa-let-7i-5p | CCR7 |
| hsa-let-7i-5p | IGF2BP1 |
| hsa-let-7i-5p | HMGA2 |
| hsa-let-7i-5p | SLC25A24 |
| hsa-let-7i-5p | C20orf112 |
| hsa-let-7i-5p | PNKD |
| hsa-let-7i-5p | FAM84B |
| hsa-let-7i-5p | HDLBP |
| hsa-let-7i-5p | CASP3 |
| hsa-let-7i-5p | NAP1L1 |
| hsa-let-7i-5p | LIPT2 |
| hsa-let-7i-5p | USP38 |
| hsa-let-7i-5p | CDC25A |
