# Supplementary material for: Testicular biopsies microarray analysis reveals circRNAs are involved in the pathogenesis of non-obstructive azoospermia
Source: Aging (Albany NY). 2020 Feb 6;12(3):2610–25. doi: 10.18632/aging.102765 (PMC7041731; doi:10.18632/aging.102765)
Supplement: Supplementary Table 2 [file aging-12-102765-s002..doc]

Supplementary Table 2.

| **miRNA** | **target** |
| --- | --- |
| hsa-miR-23c | hsa-circRNA_072697 |
| hsa-miR-23c | KDM4A |
| hsa-miR-23c | CLDN12 |
| hsa-miR-23c | PRTG |
| hsa-miR-23c | PLAU |
| hsa-miR-23c | NUFIP2 |
| hsa-miR-23c | PTEN |
| hsa-miR-23c | MSL2 |
| hsa-miR-23c | KPNA4 |
| hsa-miR-23c | FOXP2 |
| hsa-miR-23c | ATP11B |
| hsa-miR-23c | FNBP1L |
| hsa-miR-23c | ZEB1 |
| hsa-miR-23c | TIA1 |
| hsa-miR-23c | PPP1R13B |
| hsa-miR-23c | ZBTB34 |
| hsa-miR-23c | YWHAG |
| hsa-miR-23c | MET |
| hsa-miR-23c | MARCKS |
| hsa-miR-23c | TET3 |
| hsa-miR-23c | SSH2 |
| hsa-miR-23c | OSBPL8 |
| hsa-miR-23c | ANO4 |
| hsa-miR-23c | RORA |
| hsa-miR-23c | ATP6V1B2 |
| hsa-miR-23c | FMR1 |
| hsa-miR-23c | NFIA |
| hsa-miR-23c | GJA1 |
| hsa-miR-23c | LHFPL2 |
| hsa-miR-23c | STX17 |
| hsa-miR-23c | SERINC5 |
| hsa-miR-23c | CTNNBIP1 |
| hsa-miR-23c | DIP2C |
| hsa-miR-23c | HMGN2 |
| hsa-miR-23c | LAMP1 |
| hsa-miR-23c | MAP3K5 |
| hsa-miR-23c | UBE2R2 |
| hsa-miR-23c | CPEB2 |
| hsa-miR-23c | SNX27 |
| hsa-miR-23c | HIC1 |
| hsa-miR-23c | TNRC6A |
| hsa-miR-23c | VGLL3 |
| hsa-miR-23c | INTU |
| hsa-miR-23c | CEP350 |
| hsa-miR-23c | USP46 |
| hsa-miR-23c | WHSC1 |
| hsa-miR-23c | FBN2 |
| hsa-miR-23c | DCBLD2 |
| hsa-miR-23c | MAML1 |
| hsa-miR-23c | RAD23B |
| hsa-miR-23c | RTF1 |
| hsa-miR-23c | TNPO1 |
| hsa-miR-23c | LPGAT1 |
| hsa-miR-23c | HOXD10 |
| hsa-miR-23c | UBA6 |
| hsa-miR-23c | SOCS6 |
| hsa-miR-23c | SIRPA |
| hsa-miR-23c | STX12 |
| hsa-miR-23c | FAS |
| hsa-miR-23c | SLC1A1 |
| hsa-miR-23c | FAM46A |
| hsa-miR-23c | GNPDA1 |
| hsa-miR-23c | ANKHD1 |
| hsa-miR-23c | MEIS2 |
| hsa-miR-23c | IPO8 |
| hsa-miR-23c | CAMTA1 |
| hsa-miR-23c | HOOK2 |
| hsa-miR-23c | CCNG1 |
| hsa-miR-23c | BLCAP |
| hsa-miR-23c | PTP4A2 |
| hsa-miR-23c | STAT5B |
| hsa-miR-23c | SEC14L1 |
| hsa-miR-23c | DUSP5 |
| hsa-miR-23c | ERLIN2 |
| hsa-miR-23c | TET2 |
| hsa-miR-23c | TOX3 |
| hsa-miR-23c | UBE2O |
| hsa-miR-23c | C12orf76 |
| hsa-miR-23c | CELF1 |
| hsa-miR-23c | SPOPL |
| hsa-miR-23c | ZBTB2 |
| hsa-miR-23c | PPIF |
| hsa-miR-23c | FAM134C |
| hsa-miR-23c | ANKRD50 |
| hsa-miR-23c | RBM47 |
| hsa-miR-23c | SIX4 |
| hsa-miR-23c | RAI14 |
| hsa-miR-23c | JARID2 |
| hsa-miR-23c | FAM175B |
| hsa-miR-23c | MAPRE1 |
| hsa-miR-23c | PRDM10 |
| hsa-miR-23c | TXNRD1 |
| hsa-miR-23c | STK4 |
| hsa-miR-23c | CCM2 |
| hsa-miR-23c | RAB11FIP2 |
| hsa-miR-23c | PDE7A |
| hsa-miR-23c | NACC2 |
| hsa-miR-23c | KDM6A |
| hsa-miR-23c | ZNF423 |
| hsa-miR-23c | PPP1CB |
| hsa-miR-23c | CA2 |
| hsa-miR-23c | KIAA1467 |
| hsa-miR-23c | CHST7 |
| hsa-miR-23c | SEMA6D |
| hsa-miR-23c | WBP2 |
| hsa-miR-23c | IL6R |
| hsa-miR-23c | FUT4 |
| hsa-miR-23c | ZNF655 |
| hsa-miR-23c | KIAA0922 |
| hsa-miR-23c | ELF2 |
| hsa-miR-23c | MPP5 |
| hsa-miR-23c | TUSC2 |
| hsa-miR-23c | SPRY2 |
| hsa-miR-23c | GPR64 |
| hsa-miR-23c | ALDH1A2 |
| hsa-miR-23c | CAB39 |
| hsa-miR-23c | TNRC6C |
| hsa-miR-23c | CLCN3 |
| hsa-miR-23c | IRF2 |
| hsa-miR-23c | FAM126B |
| hsa-miR-23c | PBRM1 |
| hsa-miR-23c | PDE4B |
| hsa-miR-23c | SLC25A36 |
| hsa-miR-23c | PPP1R12A |
| hsa-miR-23c | ZBTB43 |
| hsa-miR-23c | MTF1 |
| hsa-miR-23c | NEK6 |
| hsa-miR-23c | SETD8 |
| hsa-miR-23c | HEXIM1 |
| hsa-miR-23c | NEK7 |
| hsa-miR-23c | TMPO |
| hsa-miR-23c | PKIA |
| hsa-miR-23c | RRAS2 |
| hsa-miR-23c | PPARGC1A |
| hsa-miR-23c | PRPF4B |
| hsa-miR-23c | VCAN |
| hsa-miR-23c | CCDC6 |
| hsa-miR-23c | GLS |
| hsa-miR-23c | GGNBP2 |
| hsa-miR-23c | PRR14L |
| hsa-miR-23c | TGIF1 |
| hsa-miR-23c | ROBO2 |
| hsa-miR-23c | ZFHX4 |
| hsa-miR-23c | CSNK1G3 |
| hsa-miR-23c | OTUB1 |
| hsa-miR-23c | TMOD1 |
| hsa-miR-23c | LRCH1 |
| hsa-miR-23c | TNKS2 |
| hsa-miR-23c | TGFBR2 |
| hsa-miR-23c | ZCCHC2 |
| hsa-miR-23c | TGFBR3 |
| hsa-miR-23c | PDIA6 |
| hsa-miR-23c | ZMYM2 |
| hsa-miR-23c | TAB3 |
| hsa-miR-23c | GXYLT1 |
| hsa-miR-23c | MARCKSL1 |
| hsa-miR-23c | TLK1 |
| hsa-miR-23c | ZNF469 |
| hsa-miR-23c | SLC38A1 |
| hsa-miR-23c | SYNJ1 |
| hsa-miR-23c | MCM3AP |
| hsa-miR-23c | SGK1 |
| hsa-miR-23c | SLC7A1 |
| hsa-miR-23c | TBC1D15 |
| hsa-miR-23c | CHST10 |
| hsa-miR-23c | BNIP3L |
| hsa-miR-23c | CHUK |
| hsa-miR-23c | HS6ST2 |
| hsa-miR-23c | PKDCC |
| hsa-miR-23c | KIAA1804 |
| hsa-miR-23c | MCFD2 |
| hsa-miR-23c | XIAP |
| hsa-miR-23c | ZFHX3 |
| hsa-miR-23c | IDH1 |
| hsa-miR-23c | NUAK2 |
| hsa-miR-23c | CFDP1 |
| hsa-miR-23c | CHSY3 |
| hsa-miR-23c | EBF3 |
| hsa-miR-23c | PKP4 |
| hsa-miR-23c | LRP5 |
| hsa-miR-23c | NPR3 |
| hsa-miR-23c | NEFL |
| hsa-miR-23c | WHAMM |
| hsa-miR-23c | ENC1 |
| hsa-miR-23c | VRK3 |
| hsa-miR-23c | ATXN7 |
| hsa-miR-23c | HAS2 |
| hsa-miR-23c | ZBTB44 |
| hsa-miR-23c | ORMDL1 |
| hsa-miR-23c | ASF1A |
| hsa-miR-23c | SAFB2 |
| hsa-miR-23c | POM121C |
| hsa-miR-23c | IPO5 |
| hsa-miR-23c | NR6A1 |
| hsa-miR-23c | SMS |
| hsa-miR-23c | NDFIP2 |
| hsa-miR-23c | IPMK |
| hsa-miR-23c | TMEM2 |
| hsa-miR-23c | MAP4K4 |
| hsa-miR-23c | DOCK3 |
| hsa-miR-23c | CASP7 |
| hsa-miR-23c | TRIB1 |
| hsa-miR-23c | LRIG1 |
| hsa-miR-23c | USP53 |
| hsa-miR-23c | RNF43 |
| hsa-miR-23c | KCNK5 |
| hsa-miR-23c | ETNK1 |
| hsa-miR-23c | RCN1 |
| hsa-miR-23c | CREBZF |
| hsa-miR-23c | WNK1 |
| hsa-miR-23c | RBM25 |
| hsa-miR-23c | C2orf69 |
| hsa-miR-23c | SEC24A |
| hsa-miR-23c | MFHAS1 |
| hsa-miR-23c | MEX3C |
| hsa-miR-23c | RBPMS2 |
| hsa-miR-23c | NAA15 |
| hsa-miR-23c | ETV1 |
| hsa-miR-23c | CCNT2 |
| hsa-miR-23c | ZNF839 |
| hsa-miR-23c | AUTS2 |
| hsa-miR-23c | GLCE |
| hsa-miR-23c | B3GNT1 |
| hsa-miR-23c | POM121 |
| hsa-miR-23c | TBC1D9 |
| hsa-miR-23c | FBN1 |
| hsa-miR-23c | FBXO32 |
| hsa-miR-23c | NUP50 |
| hsa-miR-23c | LPHN2 |
| hsa-miR-23c | SESN2 |
| hsa-miR-23c | HNRNPU |
| hsa-miR-23c | MAP3K1 |
| hsa-miR-23c | AUH |
| hsa-miR-23c | ZDBF2 |
| hsa-miR-23c | GSK3B |
| hsa-miR-23c | EGR3 |
| hsa-miR-23c | ZNF292 |
| hsa-miR-23c | TNFAIP3 |
| hsa-miR-23c | CTCF |
| hsa-miR-23c | CRLF3 |
| hsa-miR-23c | KPNA1 |
| hsa-miR-23c | INPP5A |
| hsa-miR-23c | NCOA6 |
| hsa-miR-23c | MAT2A |
| hsa-miR-23c | RPRD2 |
| hsa-miR-23c | HSPA12A |
| hsa-miR-23c | SATB1 |
| hsa-miR-23c | HMGB2 |
| hsa-miR-23c | ADAMTS6 |
| hsa-miR-23c | REEP1 |
| hsa-miR-23c | BTAF1 |
| hsa-miR-23c | PTGER4 |
| hsa-miR-23c | BRWD1 |
| hsa-miR-23c | GOLPH3L |
| hsa-miR-23c | PRDM1 |
| hsa-miR-23c | BET1 |
| hsa-miR-23c | GPBP1 |
| hsa-miR-23c | UBE2D1 |
| hsa-miR-23c | BBX |
| hsa-miR-23c | ATRN |
| hsa-miR-23c | MLLT4 |
| hsa-miR-23c | CNOT6L |
| hsa-miR-23c | ZNF652 |
| hsa-miR-23c | NAP1L1 |
| hsa-miR-23c | ENTPD5 |
| hsa-miR-23c | LBR |
| hsa-miR-23c | CBFA2T3 |
| hsa-miR-23c | TOP1 |
| hsa-miR-23c | SPOCK1 |
| hsa-miR-23c | KLF3 |
| hsa-miR-23c | DNAJC6 |
| hsa-miR-182-5p | CLOCK |
| hsa-miR-182-5p | SAMD4A |
| hsa-miR-182-5p | BCL2L12 |
| hsa-miR-182-5p | L1CAM |
| hsa-miR-182-5p | RTN4 |
| hsa-miR-182-5p | NUFIP2 |
| hsa-miR-182-5p | ADD3 |
| hsa-miR-182-5p | MBNL2 |
| hsa-miR-182-5p | TAF4B |
| hsa-miR-182-5p | CITED2 |
| hsa-miR-182-5p | FAM134B |
| hsa-miR-182-5p | MITF |
| hsa-miR-182-5p | SH3BP4 |
| hsa-miR-182-5p | EXOC4 |
| hsa-miR-182-5p | DOCK9 |
| hsa-miR-182-5p | YWHAG |
| hsa-miR-182-5p | TMEM68 |
| hsa-miR-182-5p | MARCKS |
| hsa-miR-182-5p | IGSF3 |
| hsa-miR-182-5p | FMR1 |
| hsa-miR-182-5p | CCDC117 |
| hsa-miR-182-5p | MLLT1 |
| hsa-miR-182-5p | KLF7 |
| hsa-miR-182-5p | KIAA0907 |
| hsa-miR-182-5p | ATP9A |
| hsa-miR-182-5p | DENR |
| hsa-miR-182-5p | CUL5 |
| hsa-miR-182-5p | INTS6 |
| hsa-miR-182-5p | PAIP2 |
| hsa-miR-182-5p | RNF208 |
| hsa-miR-182-5p | TACC1 |
| hsa-miR-182-5p | KPNA3 |
| hsa-miR-182-5p | PPP3R1 |
| hsa-miR-182-5p | EIF5 |
| hsa-miR-182-5p | PPIL1 |
| hsa-miR-182-5p | CCNY |
| hsa-miR-182-5p | ZFP36L1 |
| hsa-miR-182-5p | MFAP3 |
| hsa-miR-182-5p | PCMT1 |
| hsa-miR-182-5p | FRS2 |
| hsa-miR-182-5p | MYO1C |
| hsa-miR-182-5p | DCUN1D4 |
| hsa-miR-182-5p | ARRDC3 |
| hsa-miR-182-5p | ARMC1 |
| hsa-miR-182-5p | BDNF |
| hsa-miR-182-5p | LSM14A |
| hsa-miR-182-5p | ABHD13 |
| hsa-miR-182-5p | CFL1 |
| hsa-miR-182-5p | TP53INP1 |
| hsa-miR-182-5p | STARD13 |
| hsa-miR-182-5p | CAMTA1 |
| hsa-miR-182-5p | CLPTM1L |
| hsa-miR-182-5p | ARF4 |
| hsa-miR-182-5p | DCAF12 |
| hsa-miR-182-5p | PPP1R9A |
| hsa-miR-182-5p | SLCO3A1 |
| hsa-miR-182-5p | TMEM50B |
| hsa-miR-182-5p | NPM1 |
| hsa-miR-182-5p | CDC42BPA |
| hsa-miR-182-5p | SYNCRIP |
| hsa-miR-182-5p | ACVR1 |
| hsa-miR-182-5p | RECK |
| hsa-miR-182-5p | PCDH18 |
| hsa-miR-182-5p | ELMO1 |
| hsa-miR-182-5p | PC |
| hsa-miR-182-5p | SGMS2 |
| hsa-miR-182-5p | AEBP2 |
| hsa-miR-182-5p | VAMP3 |
| hsa-miR-182-5p | EVI5 |
| hsa-miR-182-5p | KCMF1 |
| hsa-miR-182-5p | TP53INP2 |
| hsa-miR-182-5p | ADCY6 |
| hsa-miR-182-5p | ZNF280B |
| hsa-miR-182-5p | NUMB |
| hsa-miR-182-5p | HOXA9 |
| hsa-miR-182-5p | AHCYL1 |
| hsa-miR-182-5p | FBXW7 |
| hsa-miR-182-5p | FBXW11 |
| hsa-miR-182-5p | FAM126B |
| hsa-miR-182-5p | FAM171A1 |
| hsa-miR-182-5p | PCNX |
| hsa-miR-182-5p | MECOM |
| hsa-miR-182-5p | CDC10 |
| hsa-miR-182-5p | SP3 |
| hsa-miR-182-5p | NUAK1 |
| hsa-miR-182-5p | MAP1B |
| hsa-miR-182-5p | KIAA1217 |
| hsa-miR-182-5p | RDX |
| hsa-miR-182-5p | ZFP36 |
| hsa-miR-182-5p | PLEKHA8 |
| hsa-miR-182-5p | CHIC1 |
| hsa-miR-182-5p | SYPL1 |
| hsa-miR-182-5p | GXYLT1 |
| hsa-miR-182-5p | SNX4 |
| hsa-miR-182-5p | BAG4 |
| hsa-miR-182-5p | MMD |
| hsa-miR-182-5p | VLDLR |
| hsa-miR-182-5p | RASA1 |
| hsa-miR-182-5p | ARHGDIA |
| hsa-miR-182-5p | SETD7 |
| hsa-miR-182-5p | CDV3 |
| hsa-miR-182-5p | N4BP1 |
| hsa-miR-182-5p | NPTX1 |
| hsa-miR-182-5p | SLC39A9 |
| hsa-miR-182-5p | RAB6B |
| hsa-miR-182-5p | CORO1C |
| hsa-miR-182-5p | CD2AP |
| hsa-miR-182-5p | TMEM145 |
| hsa-miR-182-5p | WIPI2 |
| hsa-miR-182-5p | EBF3 |
| hsa-miR-182-5p | FNDC3B |
| hsa-miR-182-5p | ADAM10 |
| hsa-miR-182-5p | TAPT1 |
| hsa-miR-182-5p | KDELR1 |
| hsa-miR-182-5p | TSNAX |
| hsa-miR-182-5p | UBE2Q2 |
| hsa-miR-182-5p | RAB10 |
| hsa-miR-182-5p | CNNM3 |
| hsa-miR-182-5p | ELL2 |
| hsa-miR-182-5p | TXNL1 |
| hsa-miR-182-5p | TAF15 |
| hsa-miR-182-5p | DMXL1 |
| hsa-miR-182-5p | DENND5B |
| hsa-miR-182-5p | FOXF2 |
| hsa-miR-182-5p | EPAS1 |
| hsa-miR-182-5p | PYGO2 |
| hsa-miR-182-5p | UBE3C |
| hsa-miR-182-5p | FOXO3 |
| hsa-miR-182-5p | PRUNE2 |
| hsa-miR-182-5p | SLC4A7 |
| hsa-miR-182-5p | SGMS1 |
| hsa-miR-182-5p | ELL |
| hsa-miR-182-5p | VAV2 |
| hsa-miR-182-5p | MAST4 |
| hsa-miR-182-5p | BNC2 |
| hsa-miR-182-5p | JAZF1 |
| hsa-miR-182-5p | FBN1 |
| hsa-miR-182-5p | LPHN2 |
| hsa-miR-182-5p | MAGI1 |
| hsa-miR-182-5p | GIT2 |
| hsa-miR-182-5p | FAM91A1 |
| hsa-miR-182-5p | KTN1 |
| hsa-miR-182-5p | CTTN |
| hsa-miR-182-5p | INSIG1 |
| hsa-miR-182-5p | RNF44 |
| hsa-miR-182-5p | LMTK2 |
| hsa-miR-182-5p | TOB1 |
| hsa-miR-182-5p | BRWD1 |
| hsa-miR-182-5p | PRDM1 |
| hsa-miR-182-5p | ZCCHC14 |
| hsa-miR-182-5p | MEF2C |
| hsa-miR-182-5p | NCKAP1 |
| hsa-miR-182-5p | WDR47 |
| hsa-miR-182-5p | hsa-circRNA_072697 |
